# (*E*)-1-[4-(Hex­yloxy)phen­yl]-3-(3-hy­droxy­phen­yl)prop-2-en-1-one

**DOI:** 10.1107/S1600536810047768

**Published:** 2010-11-24

**Authors:** Zainab Ngaini, Siti Muhaini Haris Fadzillah, Hasnain Hussain, Ibrahim Abdul Razak, Hoong-Kun Fun

**Affiliations:** a Department of Chemistry, Faculty of Resource Science and Technology, Universiti Malaysia Sarawak, 94300 Kota Samarahan, Sarawak, Malaysia; b Department of Molecular Biology, Faculty of Resource Science and Technology, Universiti Malaysia Sarawak, 94300 Kota Samarahan, Sarawak, Malaysia; c X-ray Crystallography Unit, School of Physics, Universiti Sains Malaysia, 11800 USM, Penang, Malaysia

## Abstract

There are two mol­ecules in the asymmetric unit of the title compound, C_21_H_24_O_3_, in which the dihedral angles between the aromatic rings are 6.4 (1) and 7.0 (1)°. The enone moiety of both mol­ecules adopts an *s*–*cis* configuration. In the crystal, inter­molecular O—H⋯O and C—H⋯O inter­actions to the same acceptor O atom generate *R*_2_^1^(6) ring motifs and further C—H⋯O inter­actions generate *R*_2_^2^(8) ring motifs. Topologically, the *R*_2_^1^(6) and *R*_2_^2^(8) ring motifs are arranged alternately, forming [001] chains of mol­ecules. The crystal structure is further stabilized by C—H⋯π inter­actions.

## Related literature

For general background to the biological properties of chalcone derivatives, see: Bhat *et al.* (2005 ▶); Xue *et al.* (2004 ▶); Satyanarayana *et al.* (2004 ▶); Zhao *et al.* (2005 ▶); Yayli *et al.* (2006 ▶). For related structures, see: Razak, Fun, Ngaini, Rahman *et al.* (2009 ▶); Razak, Fun, Ngaini, Fadzillah *et al.* (2009*a* ▶,*b* ▶); Ngaini, Fadzillah *et al.* (2009 ▶); Ngaini, Rahman *et al.* (2009 ▶); Razak *et al.* (2009*a* ▶,*b* ▶). For hydrogen-bond motifs, see: Bernstein *et al.* (1995 ▶). For the stability of the temperature controller used in the data collection, see: Cosier & Glazer (1986 ▶). For bond-length data, see: Allen *et al.* (1987) ▶.
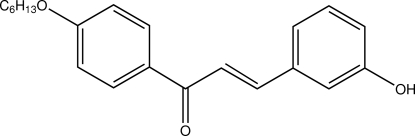


## Experimental

### 

#### Crystal data

C_21_H_24_O_3_
*M* *_r_* = 324.40
Triclinic, 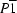

*a* = 7.6053 (3) Å
*b* = 13.7328 (5) Å
*c* = 17.3769 (7) Å
α = 105.226 (2)°
β = 93.740 (2)°
γ = 93.038 (2)°
*V* = 1742.80 (12) Å^3^
*Z* = 4
Mo *K*α radiation
μ = 0.08 mm^−1^
*T* = 100 K
0.77 × 0.44 × 0.12 mm

#### Data collection

Bruker SMART APEXII CCD diffractometer
Absorption correction: multi-scan (*SADABS*; Bruker, 2005 ▶) *T*_min_ = 0.940, *T*_max_ = 0.990
36519 measured reflections
10044 independent reflections
6371 reflections with *I* > 2σ(*I*)
*R*_int_ = 0.036

#### Refinement

*R*[*F* ^2^ > 2σ(*F* ^2^)] = 0.060
*wR*(*F* ^2^) = 0.188
*S* = 1.04
10044 reflections
443 parameters
H atoms treated by a mixture of independent and constrained refinement
Δρ_max_ = 0.66 e Å^−3^
Δρ_min_ = −0.28 e Å^−3^

### 

Data collection: *APEX2* (Bruker, 2005 ▶); cell refinement: *SAINT* (Bruker, 2005 ▶); data reduction: *SAINT*; program(s) used to solve structure: *SHELXTL* (Sheldrick, 2008 ▶); program(s) used to refine structure: *SHELXTL*; molecular graphics: *SHELXTL*; software used to prepare material for publication: *SHELXTL*.

## Supplementary Material

Crystal structure: contains datablocks global, I. DOI: 10.1107/S1600536810047768/hb5741sup1.cif

Structure factors: contains datablocks I. DOI: 10.1107/S1600536810047768/hb5741Isup2.hkl

Additional supplementary materials:  crystallographic information; 3D view; checkCIF report

## Figures and Tables

**Table 1:** Hydrogen-bond geometry (Å, °) *Cg*1 and *Cg*3 are the centroids of the C1*A*–C6*A* and C1*B*–C6*B* rings, respectively.

*D*—H⋯*A*	*D*—H	H⋯*A*	*D*⋯*A*	*D*—H⋯*A*
O1*B*—H1*OB*⋯O2*A*^i^	0.86 (3)	1.92 (3)	2.773 (2)	171 (2)
C1*B*—H1*BA*⋯O2*A*^i^	0.93	2.50	3.196 (2)	132
O1*A*—H1*OA*⋯O2*B*^ii^	0.92 (3)	1.85 (3)	2.763 (2)	175 (3)
C1*A*—H1*AA*⋯O2*B*^ii^	0.93	2.50	3.214 (2)	133
C12*B*—H12*B*⋯O3*A*^iii^	0.93	2.56	3.483 (2)	175
C12*A*—H12*A*⋯O3*B*^iv^	0.93	2.56	3.487 (2)	174
C16*A*—H16*A*⋯*Cg*1^v^	0.97	2.80	3.653 (2)	147
C16*B*—H16*C*⋯*Cg*3^vi^	0.97	2.73	3.595 (2)	149
C17*B*—H17*C*⋯*Cg*3^vii^	0.97	2.74	3.640 (2)	154

Symmetry codes: (i) 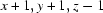; (ii) 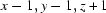; (iii) 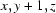; (iv) 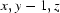; (v) 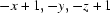; (vi) 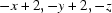; (vii) 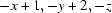.

**Fig. 1.**
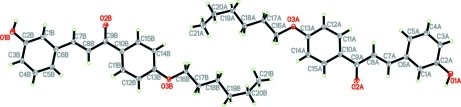
The asymmetric unit of the title compound, showing 50% probability displacement ellipsoids.

**Fig. 2.**
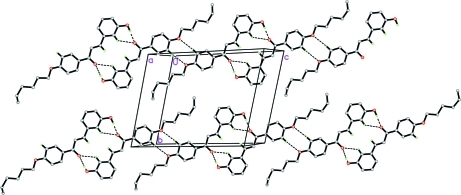
Part of the crystal packing of (I) showing R21(6) and R22(8) ring motifs generated by intermolecular interactions. The symmetry codes are given in Table 1.

## supplementary crystallographic information

### Comment

Biological properties of chalcone derivatives such as anticancer (Bhat *et al.*, 2005), antimalarial (Xue *et al.*, 2004), antioxidant and antimicrobial (Yayli *et al.*, 2006), antiplatelet (Zhao *et al.*, 2005) as well as antihyperglycemic (Satyanarayana *et al.*, 2004) activities have been widely reported. We have synthesized in our lab chalcone derivatives possessing alkyl chains which were tested against *E. coli* ATCC 8739 for their antibacterial activities. In this paper, we report one of these chalcone derivatives, the title compound (I).

There are two crystallographically independent molecules (A and B) in the asymmetric unit (Fig. 1). The bond lengths of (I) have normal values (Allen *et al.*, 1987). In molecule A, the mean plane through the enone moiety (O2/C7/C8/C9) makes a dihedral angle of 4.5 (1)° with the C1—C6 benzene ring whereas the angle is 4.4 (1)° with C10—C15 benzene ring; the corresponding angles are 8.7 (1) and 3.3 (1)°, respectively, for molecule B. The two benzene rings make a dihedral angle of 6.4 (1)° in molecule A and 7.0 (1)° in molecule B. In both molecules, the enone moiety adopts *s*-*cis* configuration with C7—C8—C9—O2 torsion angle being 3.7 (2)° in molecule A and 2.2 (2)° in molecule B.

The alkoxyl tail in both molecules are roughly coplanar with the attached benzene ring with C16—O3—C13—C14 torsion angles of 5.5 (2)° and 6.2 (2)° for molecules A and B, respectively. These chains initially maintained its planarity with the largest torsion angle deviation from the ideal 180° being 1.3 (1)° and 2.2 (1)° for O3—C16—C17—C18 in molecules A and B, respectively. However, the deviation of the alkoxyl tail from planarity starts in the aliphatic chain. The twist about the C18—C19 bond can be shown from the C17—C18—C19—C20 torsion angle of -165.1 (2)° in molecule A and -167.1 (2)° in molecule B. The twist about the C19—C20 bond are indicated by C18—C19—C20—C21 torsion angles of -67.1 (2)° for molecule A and -64.9 (2)° for molecule B.

In molecule A, the widening of C5—C6—C7 and C6—C7—C8 angles to 123.2 (2)° and 128.0 (2)°, respectively, may be the outcome of the short H5AA···H8AA (2.28 Å) contact. Similarly, strain induced through short H8AA···H11A (2.10 Å) and H14A···H16A (2.35 Å) contacts resulted in the widening of C9—C10—C11 (123.0 (2)°) and O3—C13—C14 (124.8 (2)°) angles, respectively. The distortion of the angles which is relative to what is anticipated in terms of hybridization rules can also be observed in molecule B. The opening of C5—C6—C7 (123.2 (2)°) and C6—C7—C8 (127.8 (2)°) angles is the consequence of the close interatomic contact of H5BA···H8BA (2.30 Å) while the effect of short H8BA···H11B (2.11 Å) contact resulted in the widening of C9—C10—C11 (123.2 (2)°). Likewise, the enlargement of O3—C13—C14 angle to 125.0 (2)° is due to the strain induced by short H14B···H16C (2.35 Å) contact. Similar features can also be found in related structures previously reported (Razak, Fun, Ngaini, Rahman *et al.*, 2009; Razak, Fun, Ngaini, Fadzillah *et al.*, 2009*a,b*; Ngaini, Fadzillah *et al.*, 2009; Ngaini, Rahman *et al.*, 2009).

In the crystal structure, bifurcated acceptor bond is formed by O2A atom in molecule A through O1B—H1OB···O2A^i^ and C1B—H1BA···O2A^i^ while similar acceptor bonds involving O2B in molecule B is formed through O1A—H1OA···O2B^ii^ and C1A—H1AA···O2B^ii^ intermolecular interactions (Table 1). These bifurcated acceptor bonds generate *R*_2_^1^(6) ring motifs (Bernstein *et al.*, 1995) while intermolecular C12B—H12B···O3A^iii^ and C12A—H12A···O3B^iv^ interactions involving both molecules generate an *R*_2_^2^(8) ring motifs. The *R*_2_^1^(6) and *R*_2_^2^(8) ring motifs are arranged alternately throughout the structure forming chains down on the *c*-axis (Fig. 2). The crystal structure is further stabilized by C—H···π interactions.

### Experimental

A mixture of 3-hydroxybenzaldehyde (1.22 g, 10 mmol) and 4-hexyloxyacetophenone (2.20 ml, 10 mmol) and KOH (2.02 g, 36 mmol) in 30 ml of methanol was heated at reflux for 24 h. The reaction was cooled to room temperature and acidified with cold diluted HCl (2 N). The resulting precipitate was filtered, washed and dried. After a few days of slow evaporation, colourless plates of (I) were collected.

### Refinement

All the H atoms were positioned geometrically and refined using a riding model with C—H = 0.93–0.97 Å. The *U*_iso_ values were constrained to be -1.5*U*_eq_ (methyl H atoms) and -1.2*U*_eq_ (other H atoms). The rotating model group was considered for the methyl group. In the case of O1A and O1B, the hydrogen atoms were located from a difference Fourier map and refined isotropically.

### Crystal data

**Table:**

C_21_H_24_O_3_	*Z* = 4
*M**_r_* = 324.40	*F*(000) = 696
Triclinic, *P*1	*D*_x_ = 1.236 Mg m^−^^3^
Hall symbol: -P 1	Mo *K*α radiation, λ = 0.71073 Å
*a* = 7.6053 (3) Å	Cell parameters from 9989 reflections
*b* = 13.7328 (5) Å	θ = 2.7–31.5°
*c* = 17.3769 (7) Å	µ = 0.08 mm^−^^1^
α = 105.226 (2)°	*T* = 100 K
β = 93.740 (2)°	Plate, colourless
γ = 93.038 (2)°	0.77 × 0.44 × 0.12 mm
*V* = 1742.80 (12) Å^3^	

### Data collection

**Table:**

Bruker SMART APEXII CCD diffractometer	10044 independent reflections
Radiation source: sealed tube	6371 reflections with *I* > 2σ(*I*)
graphite	*R*_int_ = 0.036
π and ω scans	θ_max_ = 30.0°, θ_min_ = 1.2°
Absorption correction: multi-scan (*SADABS*; Bruker, 2005)	*h* = −10→10
*T*_min_ = 0.940, *T*_max_ = 0.990	*k* = −19→19
36519 measured reflections	*l* = −24→23

### Refinement

**Table:**

Refinement on *F*^2^	Primary atom site location: structure-invariant direct methods
Least-squares matrix: full	Secondary atom site location: difference Fourier map
*R*[*F*^2^ > 2σ(*F*^2^)] = 0.060	Hydrogen site location: inferred from neighbouring sites
*wR*(*F*^2^) = 0.188	H atoms treated by a mixture of independent and constrained refinement
*S* = 1.03	*w* = 1/[σ^2^(*F*_o_^2^) + (0.0965*P*)^2^ + 0.6952*P*] where *P* = (*F*_o_^2^ + 2*F*_c_^2^)/3
10044 reflections	(Δ/σ)_max_ < 0.001
443 parameters	Δρ_max_ = 0.66 e Å^−^^3^
0 restraints	Δρ_min_ = −0.28 e Å^−^^3^

### Special details

**Table:**

Experimental. The crystal was placed in the cold stream of an Oxford Cryosystems Cobra open-flow nitrogen cryostat (Cosier & Glazer, 1986) operating at 100.0 (1) K.
Geometry. All e.s.d.'s (except the e.s.d. in the dihedral angle between two l.s. planes) are estimated using the full covariance matrix. The cell e.s.d.'s are taken into account individually in the estimation of e.s.d.'s in distances, angles and torsion angles; correlations between e.s.d.'s in cell parameters are only used when they are defined by crystal symmetry. An approximate (isotropic) treatment of cell e.s.d.'s is used for estimating e.s.d.'s involving l.s. planes.
Refinement. Refinement of *F*^2^ against ALL reflections. The weighted *R*-factor *wR* and goodness of fit *S* are based on *F*^2^, conventional *R*-factors *R* are based on *F*, with *F* set to zero for negative *F*^2^. The threshold expression of *F*^2^ > σ(*F*^2^) is used only for calculating *R*-factors(gt) *etc*. and is not relevant to the choice of reflections for refinement. *R*-factors based on *F*^2^ are statistically about twice as large as those based on *F*, and *R*- factors based on ALL data will be even larger.

### Fractional atomic coordinates and isotropic or equivalent isotropic displacement parameters (Å^2^)

**Table:**

	*x*	*y*	*z*	*U*_iso_*/*U*_eq_	
O1A	−0.04558 (17)	−0.32446 (10)	0.74767 (8)	0.0222 (3)	
O2A	0.17874 (17)	0.08611 (9)	0.57096 (7)	0.0229 (3)	
O3A	0.49336 (16)	0.12151 (8)	0.24926 (7)	0.0193 (3)	
C1A	0.0626 (2)	−0.22051 (13)	0.66575 (10)	0.0177 (3)	
H1AA	0.0344	−0.1623	0.7027	0.021*	
C2A	0.0299 (2)	−0.31450 (13)	0.68046 (10)	0.0181 (3)	
C3A	0.0749 (2)	−0.40150 (13)	0.62610 (11)	0.0214 (4)	
H3AA	0.0551	−0.4644	0.6360	0.026*	
C4A	0.1498 (2)	−0.39383 (13)	0.55681 (11)	0.0226 (4)	
H4AA	0.1800	−0.4521	0.5204	0.027*	
C5A	0.1801 (2)	−0.30062 (13)	0.54114 (11)	0.0204 (4)	
H5AA	0.2286	−0.2967	0.4942	0.025*	
C6A	0.1373 (2)	−0.21258 (12)	0.59617 (10)	0.0173 (3)	
C7A	0.1628 (2)	−0.11143 (13)	0.58357 (10)	0.0185 (3)	
H7AA	0.1380	−0.0577	0.6257	0.022*	
C8A	0.2170 (2)	−0.08706 (12)	0.51938 (11)	0.0177 (3)	
H8AA	0.2481	−0.1376	0.4763	0.021*	
C9A	0.2285 (2)	0.01929 (12)	0.51567 (10)	0.0161 (3)	
C10A	0.2985 (2)	0.04561 (12)	0.44537 (10)	0.0157 (3)	
C11A	0.3666 (2)	−0.02568 (12)	0.38269 (10)	0.0170 (3)	
H11A	0.3692	−0.0926	0.3847	0.020*	
C12A	0.4293 (2)	0.00219 (12)	0.31843 (10)	0.0178 (3)	
H12A	0.4733	−0.0459	0.2773	0.021*	
C13A	0.4270 (2)	0.10263 (12)	0.31485 (10)	0.0160 (3)	
C14A	0.3598 (2)	0.17511 (12)	0.37613 (10)	0.0174 (3)	
H14A	0.3576	0.2420	0.3739	0.021*	
C15A	0.2965 (2)	0.14575 (12)	0.44015 (10)	0.0177 (3)	
H15A	0.2513	0.1938	0.4809	0.021*	
C16A	0.5117 (2)	0.22438 (12)	0.24321 (11)	0.0180 (3)	
H16A	0.5771	0.2680	0.2906	0.022*	
H16B	0.3967	0.2500	0.2370	0.022*	
C17A	0.6112 (2)	0.21984 (12)	0.17017 (10)	0.0176 (3)	
H17A	0.5423	0.1760	0.1239	0.021*	
H17B	0.7215	0.1893	0.1765	0.021*	
C18A	0.6523 (2)	0.32204 (13)	0.15368 (11)	0.0205 (4)	
H18A	0.7172	0.3677	0.2003	0.025*	
H18B	0.5429	0.3514	0.1433	0.025*	
C19A	0.7623 (2)	0.30938 (13)	0.08122 (11)	0.0212 (4)	
H19A	0.8531	0.2637	0.0855	0.025*	
H19B	0.6862	0.2780	0.0331	0.025*	
C20A	0.8507 (3)	0.40815 (13)	0.07228 (12)	0.0250 (4)	
H20A	0.9201	0.4423	0.1218	0.030*	
H20B	0.9308	0.3924	0.0302	0.030*	
C21A	0.7195 (3)	0.47918 (15)	0.05250 (15)	0.0370 (5)	
H21A	0.7821	0.5388	0.0463	0.056*	
H21B	0.6432	0.4978	0.0951	0.056*	
H21C	0.6500	0.4459	0.0036	0.056*	
O1B	1.03690 (18)	1.26874 (10)	−0.36389 (8)	0.0221 (3)	
O2B	0.92123 (17)	0.87058 (9)	−0.16144 (8)	0.0234 (3)	
O3B	0.58649 (17)	0.83300 (9)	0.15508 (7)	0.0211 (3)	
C1B	0.9824 (2)	1.17003 (12)	−0.27030 (10)	0.0167 (3)	
H1BA	1.0148	1.1119	−0.3061	0.020*	
C2B	0.9882 (2)	1.26135 (12)	−0.29143 (10)	0.0167 (3)	
C3B	0.9418 (2)	1.34888 (12)	−0.23752 (11)	0.0183 (3)	
H3BA	0.9450	1.4101	−0.2511	0.022*	
C4B	0.8907 (2)	1.34372 (13)	−0.16302 (11)	0.0192 (4)	
H4BA	0.8612	1.4022	−0.1268	0.023*	
C5B	0.8830 (2)	1.25322 (13)	−0.14194 (10)	0.0182 (3)	
H5BA	0.8477	1.2511	−0.0921	0.022*	
C6B	0.9286 (2)	1.16495 (12)	−0.19592 (10)	0.0164 (3)	
C7B	0.9226 (2)	1.06597 (12)	−0.17862 (10)	0.0182 (3)	
H7BA	0.9728	1.0146	−0.2148	0.022*	
C8B	0.8539 (2)	1.04076 (12)	−0.11739 (10)	0.0175 (3)	
H8BA	0.8028	1.0893	−0.0792	0.021*	
C9B	0.8582 (2)	0.93598 (12)	−0.10954 (10)	0.0165 (3)	
C10B	0.7870 (2)	0.90971 (12)	−0.03963 (10)	0.0156 (3)	
C11B	0.7199 (2)	0.98105 (12)	0.02337 (11)	0.0176 (3)	
H11B	0.7202	1.0483	0.0221	0.021*	
C12B	0.6538 (2)	0.95274 (12)	0.08697 (11)	0.0184 (3)	
H12B	0.6102	1.0008	0.1282	0.022*	
C13B	0.6525 (2)	0.85191 (12)	0.08946 (10)	0.0168 (3)	
C14B	0.7172 (2)	0.77927 (12)	0.02725 (10)	0.0169 (3)	
H14B	0.7155	0.7119	0.0284	0.020*	
C15B	0.7835 (2)	0.80879 (12)	−0.03580 (10)	0.0170 (3)	
H15B	0.8270	0.7605	−0.0769	0.020*	
C16B	0.5958 (2)	0.73389 (12)	0.16826 (10)	0.0171 (3)	
H16C	0.7150	0.7123	0.1638	0.020*	
H16D	0.5163	0.6847	0.1294	0.020*	
C17B	0.5415 (2)	0.74426 (12)	0.25178 (10)	0.0169 (3)	
H17C	0.4226	0.7665	0.2545	0.020*	
H17D	0.6192	0.7965	0.2888	0.020*	
C18B	0.5463 (2)	0.64715 (12)	0.27882 (10)	0.0178 (3)	
H18C	0.6643	0.6236	0.2758	0.021*	
H18D	0.4655	0.5950	0.2435	0.021*	
C19B	0.4938 (2)	0.66626 (13)	0.36472 (11)	0.0203 (4)	
H19C	0.5558	0.7288	0.3968	0.024*	
H19D	0.3683	0.6756	0.3647	0.024*	
C20B	0.5329 (3)	0.58199 (14)	0.40434 (12)	0.0244 (4)	
H20C	0.6577	0.5709	0.4029	0.029*	
H20D	0.5080	0.6038	0.4601	0.029*	
C21B	0.4270 (3)	0.48291 (15)	0.36512 (14)	0.0372 (5)	
H21D	0.4515	0.4347	0.3950	0.056*	
H21E	0.4590	0.4574	0.3114	0.056*	
H21F	0.3032	0.4937	0.3642	0.056*	
H1OB	1.074 (3)	1.212 (2)	−0.3885 (16)	0.045 (7)*	
H1OA	−0.063 (4)	−0.261 (2)	0.7782 (18)	0.062 (9)*	

### Atomic displacement parameters (Å^2^)

**Table:**

	*U*^11^	*U*^22^	*U*^33^	*U*^12^	*U*^13^	*U*^23^
O1A	0.0294 (7)	0.0205 (6)	0.0194 (7)	0.0003 (5)	0.0103 (5)	0.0085 (5)
O2A	0.0319 (7)	0.0226 (6)	0.0166 (6)	0.0068 (5)	0.0104 (5)	0.0066 (5)
O3A	0.0281 (6)	0.0133 (5)	0.0186 (6)	0.0005 (5)	0.0095 (5)	0.0064 (5)
C1A	0.0193 (8)	0.0183 (8)	0.0157 (8)	0.0027 (6)	0.0036 (7)	0.0042 (6)
C2A	0.0165 (8)	0.0234 (8)	0.0166 (8)	0.0005 (6)	0.0046 (7)	0.0084 (7)
C3A	0.0251 (9)	0.0183 (8)	0.0226 (9)	0.0005 (7)	0.0050 (7)	0.0082 (7)
C4A	0.0278 (9)	0.0188 (8)	0.0213 (9)	0.0044 (7)	0.0076 (7)	0.0036 (7)
C5A	0.0248 (9)	0.0226 (8)	0.0161 (8)	0.0035 (7)	0.0071 (7)	0.0074 (7)
C6A	0.0177 (8)	0.0205 (8)	0.0149 (8)	0.0013 (6)	0.0016 (6)	0.0069 (6)
C7A	0.0208 (8)	0.0200 (8)	0.0162 (8)	0.0035 (6)	0.0044 (7)	0.0063 (6)
C8A	0.0181 (8)	0.0178 (8)	0.0174 (8)	0.0023 (6)	0.0047 (7)	0.0041 (6)
C9A	0.0145 (7)	0.0189 (8)	0.0153 (8)	0.0018 (6)	0.0012 (6)	0.0050 (6)
C10A	0.0146 (7)	0.0183 (8)	0.0148 (8)	0.0011 (6)	0.0018 (6)	0.0055 (6)
C11A	0.0196 (8)	0.0142 (7)	0.0175 (8)	0.0016 (6)	0.0027 (7)	0.0044 (6)
C12A	0.0218 (8)	0.0147 (7)	0.0163 (8)	0.0022 (6)	0.0051 (7)	0.0020 (6)
C13A	0.0150 (7)	0.0181 (8)	0.0154 (8)	−0.0005 (6)	0.0024 (6)	0.0052 (6)
C14A	0.0188 (8)	0.0153 (7)	0.0191 (9)	0.0009 (6)	0.0040 (7)	0.0060 (6)
C15A	0.0189 (8)	0.0164 (8)	0.0170 (8)	0.0023 (6)	0.0030 (7)	0.0026 (6)
C16A	0.0211 (8)	0.0141 (7)	0.0202 (9)	0.0004 (6)	0.0040 (7)	0.0069 (6)
C17A	0.0205 (8)	0.0166 (8)	0.0174 (8)	−0.0009 (6)	0.0031 (7)	0.0074 (6)
C18A	0.0257 (9)	0.0183 (8)	0.0197 (9)	−0.0003 (7)	0.0049 (7)	0.0084 (7)
C19A	0.0253 (9)	0.0193 (8)	0.0206 (9)	−0.0003 (7)	0.0061 (7)	0.0072 (7)
C20A	0.0312 (10)	0.0228 (9)	0.0217 (9)	−0.0054 (7)	0.0058 (8)	0.0076 (7)
C21A	0.0494 (13)	0.0252 (10)	0.0437 (13)	0.0081 (9)	0.0132 (11)	0.0185 (9)
O1B	0.0327 (7)	0.0212 (6)	0.0153 (6)	0.0026 (5)	0.0076 (5)	0.0087 (5)
O2B	0.0343 (7)	0.0193 (6)	0.0187 (7)	0.0042 (5)	0.0120 (6)	0.0062 (5)
O3B	0.0329 (7)	0.0158 (6)	0.0195 (6)	0.0054 (5)	0.0142 (5)	0.0096 (5)
C1B	0.0182 (8)	0.0162 (7)	0.0160 (8)	0.0008 (6)	0.0042 (6)	0.0042 (6)
C2B	0.0177 (8)	0.0202 (8)	0.0138 (8)	−0.0003 (6)	0.0023 (6)	0.0073 (6)
C3B	0.0213 (8)	0.0156 (8)	0.0200 (9)	−0.0007 (6)	0.0032 (7)	0.0082 (6)
C4B	0.0210 (8)	0.0167 (8)	0.0198 (9)	0.0007 (6)	0.0047 (7)	0.0040 (6)
C5B	0.0202 (8)	0.0208 (8)	0.0149 (8)	0.0003 (6)	0.0058 (7)	0.0065 (6)
C6B	0.0155 (7)	0.0189 (8)	0.0163 (8)	−0.0001 (6)	0.0028 (6)	0.0074 (6)
C7B	0.0219 (8)	0.0171 (8)	0.0167 (8)	0.0013 (6)	0.0052 (7)	0.0057 (6)
C8B	0.0213 (8)	0.0169 (8)	0.0159 (8)	0.0013 (6)	0.0052 (7)	0.0062 (6)
C9B	0.0171 (8)	0.0186 (8)	0.0148 (8)	−0.0002 (6)	0.0027 (6)	0.0058 (6)
C10B	0.0152 (7)	0.0181 (8)	0.0152 (8)	−0.0002 (6)	0.0025 (6)	0.0072 (6)
C11B	0.0207 (8)	0.0150 (7)	0.0188 (8)	0.0028 (6)	0.0040 (7)	0.0067 (6)
C12B	0.0231 (8)	0.0158 (8)	0.0181 (9)	0.0045 (6)	0.0073 (7)	0.0056 (6)
C13B	0.0164 (8)	0.0191 (8)	0.0170 (8)	0.0009 (6)	0.0048 (6)	0.0075 (6)
C14B	0.0192 (8)	0.0144 (7)	0.0193 (8)	0.0022 (6)	0.0061 (7)	0.0071 (6)
C15B	0.0184 (8)	0.0171 (8)	0.0163 (8)	0.0024 (6)	0.0040 (6)	0.0053 (6)
C16B	0.0208 (8)	0.0149 (7)	0.0179 (8)	0.0021 (6)	0.0053 (7)	0.0077 (6)
C17B	0.0196 (8)	0.0162 (8)	0.0162 (8)	0.0008 (6)	0.0054 (6)	0.0060 (6)
C18B	0.0224 (8)	0.0172 (8)	0.0166 (8)	0.0018 (6)	0.0066 (7)	0.0080 (6)
C19B	0.0261 (9)	0.0193 (8)	0.0175 (9)	0.0018 (7)	0.0054 (7)	0.0076 (7)
C20B	0.0301 (10)	0.0255 (9)	0.0228 (9)	0.0053 (7)	0.0081 (8)	0.0137 (7)
C21B	0.0598 (15)	0.0233 (10)	0.0336 (12)	−0.0012 (9)	0.0109 (11)	0.0155 (9)

### Geometric parameters (Å, °)

**Table:**

O1A—C2A	1.369 (2)	O1B—C2B	1.363 (2)
O1A—H1OA	0.92 (3)	O1B—H1OB	0.85 (3)
O2A—C9A	1.237 (2)	O2B—C9B	1.235 (2)
O3A—C13A	1.355 (2)	O3B—C13B	1.355 (2)
O3A—C16A	1.4440 (19)	O3B—C16B	1.4428 (19)
C1A—C2A	1.393 (2)	C1B—C2B	1.395 (2)
C1A—C6A	1.396 (2)	C1B—C6B	1.399 (2)
C1A—H1AA	0.9300	C1B—H1BA	0.9300
C2A—C3A	1.389 (2)	C2B—C3B	1.394 (2)
C3A—C4A	1.391 (3)	C3B—C4B	1.394 (2)
C3A—H3AA	0.9300	C3B—H3BA	0.9300
C4A—C5A	1.388 (2)	C4B—C5B	1.384 (2)
C4A—H4AA	0.9300	C4B—H4BA	0.9300
C5A—C6A	1.399 (2)	C5B—C6B	1.400 (2)
C5A—H5AA	0.9300	C5B—H5BA	0.9300
C6A—C7A	1.467 (2)	C6B—C7B	1.466 (2)
C7A—C8A	1.330 (2)	C7B—C8B	1.331 (2)
C7A—H7AA	0.9300	C7B—H7BA	0.9300
C8A—C9A	1.477 (2)	C8B—C9B	1.482 (2)
C8A—H8AA	0.9300	C8B—H8BA	0.9300
C9A—C10A	1.484 (2)	C9B—C10B	1.481 (2)
C10A—C15A	1.403 (2)	C10B—C15B	1.404 (2)
C10A—C11A	1.407 (2)	C10B—C11B	1.408 (2)
C11A—C12A	1.377 (2)	C11B—C12B	1.379 (2)
C11A—H11A	0.9300	C11B—H11B	0.9300
C12A—C13A	1.398 (2)	C12B—C13B	1.396 (2)
C12A—H12A	0.9300	C12B—H12B	0.9300
C13A—C14A	1.398 (2)	C13B—C14B	1.400 (2)
C14A—C15A	1.384 (2)	C14B—C15B	1.379 (2)
C14A—H14A	0.9300	C14B—H14B	0.9300
C15A—H15A	0.9300	C15B—H15B	0.9300
C16A—C17A	1.509 (2)	C16B—C17B	1.508 (2)
C16A—H16A	0.9700	C16B—H16C	0.9700
C16A—H16B	0.9700	C16B—H16D	0.9700
C17A—C18A	1.525 (2)	C17B—C18B	1.527 (2)
C17A—H17A	0.9700	C17B—H17C	0.9700
C17A—H17B	0.9700	C17B—H17D	0.9700
C18A—C19A	1.534 (2)	C18B—C19B	1.529 (2)
C18A—H18A	0.9700	C18B—H18C	0.9700
C18A—H18B	0.9700	C18B—H18D	0.9700
C19A—C20A	1.531 (2)	C19B—C20B	1.526 (2)
C19A—H19A	0.9700	C19B—H19C	0.9700
C19A—H19B	0.9700	C19B—H19D	0.9700
C20A—C21A	1.517 (3)	C20B—C21B	1.513 (3)
C20A—H20A	0.9700	C20B—H20C	0.9700
C20A—H20B	0.9700	C20B—H20D	0.9700
C21A—H21A	0.9600	C21B—H21D	0.9600
C21A—H21B	0.9600	C21B—H21E	0.9600
C21A—H21C	0.9600	C21B—H21F	0.9600
			
C2A—O1A—H1OA	107.6 (18)	C2B—O1B—H1OB	108.3 (18)
C13A—O3A—C16A	119.39 (13)	C13B—O3B—C16B	119.73 (13)
C2A—C1A—C6A	120.84 (16)	C2B—C1B—C6B	120.76 (15)
C2A—C1A—H1AA	119.6	C2B—C1B—H1BA	119.6
C6A—C1A—H1AA	119.6	C6B—C1B—H1BA	119.6
O1A—C2A—C3A	118.21 (15)	O1B—C2B—C3B	117.90 (15)
O1A—C2A—C1A	121.99 (15)	O1B—C2B—C1B	122.35 (15)
C3A—C2A—C1A	119.80 (16)	C3B—C2B—C1B	119.75 (15)
C2A—C3A—C4A	119.48 (16)	C4B—C3B—C2B	119.28 (15)
C2A—C3A—H3AA	120.3	C4B—C3B—H3BA	120.4
C4A—C3A—H3AA	120.3	C2B—C3B—H3BA	120.4
C5A—C4A—C3A	121.01 (16)	C5B—C4B—C3B	121.31 (16)
C5A—C4A—H4AA	119.5	C5B—C4B—H4BA	119.3
C3A—C4A—H4AA	119.5	C3B—C4B—H4BA	119.3
C4A—C5A—C6A	119.79 (16)	C4B—C5B—C6B	119.71 (16)
C4A—C5A—H5AA	120.1	C4B—C5B—H5BA	120.1
C6A—C5A—H5AA	120.1	C6B—C5B—H5BA	120.1
C1A—C6A—C5A	119.05 (15)	C1B—C6B—C5B	119.19 (15)
C1A—C6A—C7A	117.76 (15)	C1B—C6B—C7B	117.64 (15)
C5A—C6A—C7A	123.17 (16)	C5B—C6B—C7B	123.17 (15)
C8A—C7A—C6A	128.02 (16)	C8B—C7B—C6B	127.82 (16)
C8A—C7A—H7AA	116.0	C8B—C7B—H7BA	116.1
C6A—C7A—H7AA	116.0	C6B—C7B—H7BA	116.1
C7A—C8A—C9A	120.77 (16)	C7B—C8B—C9B	120.59 (16)
C7A—C8A—H8AA	119.6	C7B—C8B—H8BA	119.7
C9A—C8A—H8AA	119.6	C9B—C8B—H8BA	119.7
O2A—C9A—C8A	119.90 (16)	O2B—C9B—C10B	120.05 (15)
O2A—C9A—C10A	120.17 (15)	O2B—C9B—C8B	119.86 (15)
C8A—C9A—C10A	119.93 (14)	C10B—C9B—C8B	120.09 (14)
C15A—C10A—C11A	117.86 (16)	C15B—C10B—C11B	117.80 (15)
C15A—C10A—C9A	119.11 (15)	C15B—C10B—C9B	119.00 (15)
C11A—C10A—C9A	123.03 (15)	C11B—C10B—C9B	123.19 (15)
C12A—C11A—C10A	120.96 (15)	C12B—C11B—C10B	121.04 (15)
C12A—C11A—H11A	119.5	C12B—C11B—H11B	119.5
C10A—C11A—H11A	119.5	C10B—C11B—H11B	119.5
C11A—C12A—C13A	120.12 (15)	C11B—C12B—C13B	119.99 (15)
C11A—C12A—H12A	119.9	C11B—C12B—H12B	120.0
C13A—C12A—H12A	119.9	C13B—C12B—H12B	120.0
O3A—C13A—C12A	115.01 (14)	O3B—C13B—C12B	114.92 (14)
O3A—C13A—C14A	124.78 (15)	O3B—C13B—C14B	124.96 (15)
C12A—C13A—C14A	120.21 (16)	C12B—C13B—C14B	120.11 (16)
C15A—C14A—C13A	118.93 (15)	C15B—C14B—C13B	119.23 (15)
C15A—C14A—H14A	120.5	C15B—C14B—H14B	120.4
C13A—C14A—H14A	120.5	C13B—C14B—H14B	120.4
C14A—C15A—C10A	121.91 (15)	C14B—C15B—C10B	121.81 (15)
C14A—C15A—H15A	119.0	C14B—C15B—H15B	119.1
C10A—C15A—H15A	119.0	C10B—C15B—H15B	119.1
O3A—C16A—C17A	105.46 (13)	O3B—C16B—C17B	105.71 (13)
O3A—C16A—H16A	110.6	O3B—C16B—H16C	110.6
C17A—C16A—H16A	110.6	C17B—C16B—H16C	110.6
O3A—C16A—H16B	110.6	O3B—C16B—H16D	110.6
C17A—C16A—H16B	110.6	C17B—C16B—H16D	110.6
H16A—C16A—H16B	108.8	H16C—C16B—H16D	108.7
C16A—C17A—C18A	114.68 (14)	C16B—C17B—C18B	114.30 (14)
C16A—C17A—H17A	108.6	C16B—C17B—H17C	108.7
C18A—C17A—H17A	108.6	C18B—C17B—H17C	108.7
C16A—C17A—H17B	108.6	C16B—C17B—H17D	108.7
C18A—C17A—H17B	108.6	C18B—C17B—H17D	108.7
H17A—C17A—H17B	107.6	H17C—C17B—H17D	107.6
C17A—C18A—C19A	110.30 (14)	C17B—C18B—C19B	110.22 (14)
C17A—C18A—H18A	109.6	C17B—C18B—H18C	109.6
C19A—C18A—H18A	109.6	C19B—C18B—H18C	109.6
C17A—C18A—H18B	109.6	C17B—C18B—H18D	109.6
C19A—C18A—H18B	109.6	C19B—C18B—H18D	109.6
H18A—C18A—H18B	108.1	H18C—C18B—H18D	108.1
C20A—C19A—C18A	114.49 (15)	C20B—C19B—C18B	114.61 (14)
C20A—C19A—H19A	108.6	C20B—C19B—H19C	108.6
C18A—C19A—H19A	108.6	C18B—C19B—H19C	108.6
C20A—C19A—H19B	108.6	C20B—C19B—H19D	108.6
C18A—C19A—H19B	108.6	C18B—C19B—H19D	108.6
H19A—C19A—H19B	107.6	H19C—C19B—H19D	107.6
C21A—C20A—C19A	113.07 (16)	C21B—C20B—C19B	113.65 (16)
C21A—C20A—H20A	109.0	C21B—C20B—H20C	108.8
C19A—C20A—H20A	109.0	C19B—C20B—H20C	108.8
C21A—C20A—H20B	109.0	C21B—C20B—H20D	108.8
C19A—C20A—H20B	109.0	C19B—C20B—H20D	108.8
H20A—C20A—H20B	107.8	H20C—C20B—H20D	107.7
C20A—C21A—H21A	109.5	C20B—C21B—H21D	109.5
C20A—C21A—H21B	109.5	C20B—C21B—H21E	109.5
H21A—C21A—H21B	109.5	H21D—C21B—H21E	109.5
C20A—C21A—H21C	109.5	C20B—C21B—H21F	109.5
H21A—C21A—H21C	109.5	H21D—C21B—H21F	109.5
H21B—C21A—H21C	109.5	H21E—C21B—H21F	109.5
			
C6A—C1A—C2A—O1A	−179.04 (15)	C6B—C1B—C2B—O1B	−178.82 (15)
C6A—C1A—C2A—C3A	1.2 (3)	C6B—C1B—C2B—C3B	0.8 (2)
O1A—C2A—C3A—C4A	179.22 (15)	O1B—C2B—C3B—C4B	179.76 (15)
C1A—C2A—C3A—C4A	−1.0 (3)	C1B—C2B—C3B—C4B	0.1 (2)
C2A—C3A—C4A—C5A	−0.1 (3)	C2B—C3B—C4B—C5B	−0.8 (3)
C3A—C4A—C5A—C6A	1.0 (3)	C3B—C4B—C5B—C6B	0.5 (3)
C2A—C1A—C6A—C5A	−0.3 (2)	C2B—C1B—C6B—C5B	−1.1 (2)
C2A—C1A—C6A—C7A	178.12 (15)	C2B—C1B—C6B—C7B	178.81 (15)
C4A—C5A—C6A—C1A	−0.8 (3)	C4B—C5B—C6B—C1B	0.4 (2)
C4A—C5A—C6A—C7A	−179.09 (16)	C4B—C5B—C6B—C7B	−179.44 (16)
C1A—C6A—C7A—C8A	−173.88 (17)	C1B—C6B—C7B—C8B	−170.15 (17)
C5A—C6A—C7A—C8A	4.5 (3)	C5B—C6B—C7B—C8B	9.7 (3)
C6A—C7A—C8A—C9A	177.51 (16)	C6B—C7B—C8B—C9B	179.51 (16)
C7A—C8A—C9A—O2A	−3.7 (2)	C7B—C8B—C9B—O2B	−2.2 (3)
C7A—C8A—C9A—C10A	176.67 (15)	C7B—C8B—C9B—C10B	177.85 (15)
O2A—C9A—C10A—C15A	−4.2 (2)	O2B—C9B—C10B—C15B	−3.5 (2)
C8A—C9A—C10A—C15A	175.42 (14)	C8B—C9B—C10B—C15B	176.44 (15)
O2A—C9A—C10A—C11A	176.26 (16)	O2B—C9B—C10B—C11B	177.33 (16)
C8A—C9A—C10A—C11A	−4.1 (2)	C8B—C9B—C10B—C11B	−2.7 (2)
C15A—C10A—C11A—C12A	0.1 (2)	C15B—C10B—C11B—C12B	0.4 (2)
C9A—C10A—C11A—C12A	179.65 (15)	C9B—C10B—C11B—C12B	179.52 (15)
C10A—C11A—C12A—C13A	0.3 (2)	C10B—C11B—C12B—C13B	−0.1 (3)
C16A—O3A—C13A—C12A	−174.49 (14)	C16B—O3B—C13B—C12B	−173.44 (14)
C16A—O3A—C13A—C14A	5.5 (2)	C16B—O3B—C13B—C14B	6.2 (2)
C11A—C12A—C13A—O3A	179.47 (14)	C11B—C12B—C13B—O3B	179.27 (15)
C11A—C12A—C13A—C14A	−0.5 (2)	C11B—C12B—C13B—C14B	−0.4 (3)
O3A—C13A—C14A—C15A	−179.74 (15)	O3B—C13B—C14B—C15B	−179.00 (15)
C12A—C13A—C14A—C15A	0.2 (2)	C12B—C13B—C14B—C15B	0.6 (2)
C13A—C14A—C15A—C10A	0.2 (2)	C13B—C14B—C15B—C10B	−0.3 (2)
C11A—C10A—C15A—C14A	−0.4 (2)	C11B—C10B—C15B—C14B	−0.1 (2)
C9A—C10A—C15A—C14A	−179.95 (15)	C9B—C10B—C15B—C14B	−179.32 (15)
C13A—O3A—C16A—C17A	172.30 (13)	C13B—O3B—C16B—C17B	170.35 (14)
O3A—C16A—C17A—C18A	−177.78 (14)	O3B—C16B—C17B—C18B	−178.70 (13)
C16A—C17A—C18A—C19A	176.80 (15)	C16B—C17B—C18B—C19B	178.55 (14)
C17A—C18A—C19A—C20A	−165.05 (15)	C17B—C18B—C19B—C20B	−167.08 (15)
C18A—C19A—C20A—C21A	−67.1 (2)	C18B—C19B—C20B—C21B	−64.9 (2)

### Hydrogen-bond geometry (Å, °)

**Table:**

Cg1 and Cg3 are the centroids of the C1A–C6A and C1B–C6B rings, respectively.

**Table:**

*D*—H···*A*	*D*—H	H···*A*	*D*···*A*	*D*—H···*A*
O1B—H1OB···O2A^i^	0.86 (3)	1.92 (3)	2.773 (2)	171 (2)
C1B—H1BA···O2A^i^	0.93	2.50	3.196 (2)	132
O1A—H1OA···O2B^ii^	0.92 (3)	1.85 (3)	2.763 (2)	175 (3)
C1A—H1AA···O2B^ii^	0.93	2.50	3.214 (2)	133
C12B—H12B···O3A^iii^	0.93	2.56	3.483 (2)	175
C12A—H12A···O3B^iv^	0.93	2.56	3.487 (2)	174
C16A—H16A···Cg1^v^	0.97	2.80	3.653 (2)	147
C16B—H16C···Cg3^vi^	0.97	2.73	3.595 (2)	149
C17B—H17C···Cg3^vii^	0.97	2.74	3.640 (2)	154

Symmetry codes: (i) *x*+1, *y*+1, *z*−1; (ii) *x*−1, *y*−1, *z*+1; (iii) *x*, *y*+1, *z*; (iv) *x*, *y*−1, *z*; (v) −*x*+1, −*y*, −*z*+1; (vi) −*x*+2, −*y*+2, −*z*; (vii) −*x*+1, −*y*+2, −*z*.
